# Thromboprophylaxis After ACL Reconstruction: Controversies and Future Challenges

**DOI:** 10.3390/medicina62071315

**Published:** 2026-07-08

**Authors:** Theodoros Bouras, Panagiotis Antzoulas, Vasileios Giannatos, Ioanna Lianou, Alexandros Voutsinas Kandilioros, Dimitrios Ntourantonis, Vasileios Chouliaras

**Affiliations:** 1 Department of Orthopaedics, General Hospital of Patras, 263 32 Patras, Greece; theo_bouras@hotmail.com (T.B.); jlianou@med.uoa.gr (I.L.); alexvoutsinas.k@gmail.com (A.V.K.); 2 Department of Orthopaedics, University Hospital of Patras, 265 04 Patras, Greece; p.antzoulas@hotmail.com (P.A.); vasileiosgiannatos@outlook.com (V.G.); 3 Emergency Department, University Hospital of Patras, 265 04 Patras, Greece; 4 Department of Orthopedics, General Hospital of Arta, 471 00 Arta, Greece; vashoulio21@gmail.com

**Keywords:** ACL reconstruction, thromboprophylaxis, venous thromboembolism, risk stratification, arthroscopy, orthopaedic trauma, clinical decision-making

## Abstract

*Background and Objectives*: Anterior cruciate ligament reconstruction (ACLR) is a very common procedure in young, active individuals. One of the rarest, but potentially life-threatening complications is symptomatic venous thromboembolism (VTE). Despite that, international guidelines offer conflicting advice on routine thromboprophylaxis. This review aims to summarize key international recommendations to support clinical decision-making. *Materials and Methods*: The role of thromboprophylaxis after ACL reconstruction remains controversial. Rather than performing a systematic review, relevant documents were identified from official publications, consensus statements, and registry-based recommendations issued by recognized orthopaedic and thrombosis-related organizations. Documents were included if they represented formal guidelines, consensus statements, or national/registry-based recommendations with direct clinical relevance to ACLR and thromboprophylaxis, and if they reflected contemporary practice across different healthcare systems. Recommendations from CHEST, NICE, BOA/BASK/BOSTAA, AAOS, ICM-VTE, ICS, SFAR, HAOST, and the Swedish Knee Ligament Registry were included. These were analyzed with respect to indications for pharmacological and mechanical prophylaxis, as well as risk stratification strategies. *Results*: This narrative review identified nine major international guidelines addressing thromboprophylaxis in ACLR. Most guidelines, including CHEST and NICE, advise against routine anticoagulation for low-risk patients, reserving it for those with specific risk factors. BOA recommends prophylaxis only when multiple comorbidities are present. The Swedish registry indicates that anticoagulation is primarily used in patients with prior VTE or oral contraceptive use. While ICM-VTE supports the use of aspirin or LMWH in high-risk cases, SFAR stands out by recommending routine LMWH for all patients. HAOST emphasizes early mobilization and selective prophylaxis, including aspirin for low-risk groups. *Conclusions*: Current evidence does not support universal thromboprophylaxis following ACLR. A risk-stratified approach is recommended, with mechanical measures or aspirin for low-to-moderate-risk patients and LMWH or DOACs for high-risk individuals. Future research may focus on genetic and patient-specific factors to better explain existing heterogeneity.

## 1. Introduction

Venous thromboembolism (VTE), including deep vein thrombosis (DVT) and pulmonary embolism (PE), remains one of the most important causes of morbidity and mortality in orthopaedics [1]. Thromboprophylaxis is essential for perioperative care in high- risk populations, mainly in patients undergoing total hip or knee arthroplasty, where the use of pharmacologic and mechanical strategies has efficacy in decreasing VTE-related complications [2].

Anterior cruciate ligament reconstruction (ACLR) is one of the most commonly performed procedures in arthroscopic surgery, with more than 100,000 procedures performed annually in the United States, and is typically undertaken following acute trauma in young, physically active individuals, often in combination with postoperative weight-bearing restrictions, brace immobilization, and structured rehabilitation protocols [1]. These characteristics introduce a distinct thromboembolic risk profile that cannot be directly extrapolated from data derived from simple diagnostic knee arthroscopy [2]. The venous thromboembolism (VTE) rate after ACLR is low in the general population (0.3–1.1%), but higher among subgroups with risk factors, such as a prior history of VTE, known thrombophilia, obesity, prolonged immobilization, estrogen use, advanced age (>60 years), and comorbidities [3,4]. Furthermore, the use of a tourniquet, graft harvesting, and concomitant procedures such as meniscal repair may prolong operative time and postoperative immobility, contributing to endothelial injury and venous stasis [1,2]. As a result, ACLR occupies a “grey zone” between low-risk arthroscopic procedures and major lower-limb orthopedic surgery with regard to venous thromboembolism risk.

Even today, the role of thromboprophylaxis in arthroscopic procedures—and specifically in ACLR—remains controversial. The main challenge is the balancing of low risk of VTE against the complications of anticoagulant use, particularly postoperative bleeding and arthrofibrosis [5]. The existing international guidelines differ in philosophy, levels of evidence, and strength of recommendations. Some of them advocate routine prophylaxis for all patients undergoing ACLR; others recommend a risk-stratified approach for those with established predisposing factors such as prior VTE, thrombophilia, prolonged immobilization, obesity, or estrogen therapy [6,7]. Pharmacologic prophylaxis has been linked to an increased incidence of arthrofibrosis and the need for manipulation under anesthesia (MUA) or lysis of adhesions (LOA), especially in young and active patients [8].

In this context, the absence of uniform international recommendations regarding thromboprophylaxis after ACLR reflects the ongoing controversy between preventing rare thromboembolic events and avoiding overtreatment. A trauma-specific, risk-adapted approach appears increasingly relevant, emphasizing individualized assessment rather than routine anticoagulation.

The aim of this narrative review is to summarize and compare the recommendations of selected international guidelines and consensus statements regarding thromboprophylaxis following ACLR, and to discuss the clinical implications for risk-based patient management. Furthermore, it seeks to address the clinical question of whether routine pharmacological thromboprophylaxis should be administered in patients undergoing ACLR following traumatic ACL injury.

## 2. Materials and Methods

This study was conducted as a narrative review of international guidelines and consensus statements addressing thromboprophylaxis following ACLR. Documents were selected based on clinical relevance, representation of contemporary practice, and inclusion of formal guideline or consensus recommendations from recognized organizations.

Our aim was not to perform a systematic quantitative synthesis, but to provide a structured overview of current recommendations across different healthcare systems and official registries. The included documents were selected based on their clinical relevance and representation of contemporary practice.

All documents were narratively analyzed with regard to recommendations for routine thromboprophylaxis, indications for pharmacological prophylaxis, suggested agents and duration, and the use of risk-stratification strategies.

Additional key observational studies and systematic reviews were considered to provide clinical context regarding thromboembolic risk and complications such as arthrofibrosis.

## 3. Results

Comparative analysis of nine documents—comprising formal guidelines, consensus statements, and national or registry-based recommendations—revealed significant heterogeneity in thromboprophylaxis strategies following ACLR.

Specifically, the reviewed documents were categorized as follows:

- Formal guidelines: the American College of Chest Physicians (CHEST) [1,9], the National Institute for Health and Care Excellence (NICE) [6], the American Academy of Orthopaedic Surgeons (AAOS) [7,10], and the Société Française d’Anesthésie et de Réanimation (SFAR) [11].
- Consensus statements: the International Consensus Meeting on Venous Thromboembolism: Sports (ICM-VTE: Sports) [12], the International Consensus Statement on Venous Thromboembolism (ICS) [13], and the British Orthopaedic Association in collaboration with the British Association for Surgery of the Knee and BOSTAA (BOA/BASK/BOSTAA) [14].
- National or registry-based recommendations: the Hellenic Association of Orthopaedic Surgery and Traumatology (HAOST) [15] and data from the Swedish Knee Ligament Registry [16].

A consistent finding across several formal guidelines and consensus statements is that ACLR is generally considered a low-risk procedure for VTE in otherwise healthy and mobile individuals [9,12,13,14]. Consequently, routine pharmacological thromboprophylaxis is not recommended, and a risk-stratified approach is favored [6,9,12,14].

This position is supported by CHEST, NICE, BOA/BASK in collaboration with the UK Haemophilia Centre Doctors’ Organisation (UKHCDO), ICS, and ICM-VTE [6,9,12,13,14]. These documents emphasize that thromboprophylaxis should be reserved for patients with additional risk factors, such as prior VTE, thrombophilia, prolonged immobilization, obesity, or hormonal therapy use [9,12,14].

Risk stratification tools, such as the Caprini score, are frequently recommended to guide decision-making, particularly by BOA/BASK [14,17]. Early mobilization is consistently highlighted as a key preventive measure across these recommendations [9,13,14].

Some variations exist within this group. NICE suggests considering thromboprophylaxis in procedures lasting more than 90 min or in the presence of additional risk factors [6]. The ICS recommends pharmacological prophylaxis or mechanical methods such as intermittent pneumatic compression (IPC) in more complex procedures, including ligament reconstruction [18]. The ICM-VTE consensus further supports the use of LMWH, aspirin, or DOACs in selected high-risk patients, based on moderate- to low-quality evidence, without a clear difference in bleeding risk among agents [12].

In contrast, the SFAR has maintained, since 2011, LMWH prophylaxis for at least 10 days after ACL reconstruction for all patients, regardless of risk profile [11]. This recommendation lacks detailed risk stratification, and it is not supported by studies comparing efficacy against complication rates. Other French professional societies, such as the French Orthopaedic Society (Société Française d’Orthopédie), have expressed reservations about this routine approach [11]. The AAOS, in its 2022 Clinical Practice Guideline (CPG) for ACL injury management, does not report a specific recommendation regarding thromboprophylaxis following reconstruction [7]. Although postoperative complications are discussed, the guideline does not provide clear advice about using LMWH or DOAC [7]. This absence of guidelines reflects limited supporting evidence rather than disagreements against prophylaxis. The AAOS emphasizes early mobilization and functional recovery, leaving the decision for anticoagulant use to the physician, based on individualized risk–benefit assessment.

Finally, HAOST, in its 2009 guidelines on the prevention of venous thromboembolism in orthopaedics, reports that studies of knee arthroscopy without thromboprophylaxis demonstrated a VTE incidence ranging from 5% to 9%, with above-knee DVT rates between 0.7% and 3% [15]. The risk of VTE is higher in therapeutic procedures, such as ACLR, compared with simple diagnostic arthroscopy, due to increased procedural complexity, longer operative time, postoperative immobilization, and the use of a tourniquet [15].

HAOST does not recommend routine pharmacological thromboprophylaxis but emphasizes early and frequent mobilization. In patients with additional risk factors or undergoing more complex arthroscopic procedures, the use of LMWH or mechanical prophylaxis (in cases where LMWH is contraindicated) is recommended until full mobilization is achieved [15].

## 4. Discussion

International guidelines demonstrate a lack of universal consensus regarding thromboprophylaxis following anterior cruciate ligament reconstruction (ACLR) [6,9,12,13,14]. Most guidelines—including those from the American College of Chest Physicians (CHEST), the National Institute for Health and Care Excellence (NICE), the British Orthopaedic Association (BOA), the International Consensus Meeting on VTE (ICM-VTE), and the International Consensus Statement (ICS)—do not suggest routine pharmacological thromboprophylaxis and emphasize the importance of individualized risk stratification [6,9,12,13,14].

In contrast, other organizations, such as the American Academy of Orthopaedic Surgeons (AAOS) and the Hellenic Association of Orthopaedic Surgery and Traumatology (HAOST), provide limited or inconclusive recommendations, reflecting the lack of high-quality evidence in this field [7,15]. The Société Française d’Anesthésie et de Réanimation (SFAR) represents a notable exception, as it recommends routine administration of low-molecular-weight heparin (LMWH) for at least 10 days following ACL reconstruction, regardless of patient risk profile [11].

An important limitation of the current guideline landscape is the quality of the underlying evidence. Many recommendations are derived from expert consensus, extrapolation from broader orthopaedic populations, or studies including heterogeneous arthroscopic procedures rather than ACL reconstruction specifically. Consequently, the strength of evidence supporting many recommendations remains limited, highlighting the need for ACL-specific prospective studies and randomized controlled trials.

A key factor underlying this lack of consensus might be the delicate balance between preventing rare thromboembolic events and avoiding complications that may compromise postoperative recovery. Arthrofibrosis represents one of the most clinically relevant complications following ACL reconstruction, as it directly affects range of motion, rehabilitation progress, and long-term functional outcomes [8]. Several observational studies have reported an association between pharmacologic thromboprophylaxis and postoperative stiffness, manipulation under anesthesia, or lysis of adhesions following ACL reconstruction. However, the currently available evidence is predominantly retrospective and observational, and residual confounding cannot be excluded. Therefore, a definitive causal relationship has not yet been established [8]. Consequently, the potential benefit of reducing VTE incidence must be carefully weighed against the risk of impairing knee function, particularly in young and athletic populations where optimal recovery is paramount.

Brophy and Lowry report that thromboprophylaxis after ACLR is not a strong recommendation due to insufficient high-level data on its effect [10]. Supporting this position, the systematic review by Bayle-Iñiguez et al. found that the incidence of symptomatic DVT is low even without the use of LMWH, raising concerns against routine thromboprophylaxis, particularly in younger patients with a low risk profile [18].

Observational data from clinical practice further support a risk-based approach. A study by Ekdahl et al. in Sweden demonstrated that only 18% of surgeons routinely prescribe thromboprophylaxis following ACL reconstruction [16]. The majority of orthopedic surgeons favor individualized, risk-based decision-making, reserving pharmacological thromboprophylaxis for patients with additional risk factors such as oral contraceptive use, prior VTE, or limited weight-bearing [16]. This approach aligns with the principle of avoiding overtreatment and is supported by the low reported incidence of VTE (0.3–0.5%) in the Swedish population undergoing ACLR [16].

Another source of controversy is the discrepancy between symptomatic and asymptomatic thromboembolic events. Although symptomatic VTE rates after ACL reconstruction remain relatively low, studies utilizing routine postoperative ultrasonographic screening have reported substantially higher rates of asymptomatic deep vein thrombosis. The clinical significance of these findings remains uncertain, as many asymptomatic thrombi may not progress to clinically relevant events. Nevertheless, these observations suggest that the true thrombotic burden after ACL reconstruction may be underestimated when only symptomatic events are considered.

The Caprini Risk Assessment Model remains the most widely adopted tool for thromboembolic risk stratification, but it has not been specifically validated for ACL reconstruction [17]. According to the Caprini scoring system, patients with a score ≥ 3 are classified as high risk and are candidates for pharmacologic prophylaxis [17]. However, in young, healthy individuals without additional risk factors, Caprini scores are typically low (0–1), and routine LMWH or DOAC use is generally not recommended [17]. Although not officially validated for thromboprophylaxis in ACLR, the guidelines of the British Orthopaedic Association (BOA) include its use for decision-making, suggesting the usual cutoff of ≥3 as an indication for pharmacological thromboprophylaxis [17]. Ye et al. in 2013 observed that female gender and age > 35 years could also be high-risk factors for VTE development [19]. Other studies have also validated similar risk factors including period of non-weight bearing, tourniquet time, tobacco use, chronic obstructive pulmonary disease (COPD), oral contraceptives, concomitant meniscal procedures and high BMI [4,20,21,22].

An additional consideration is the influence of procedure-specific factors on thromboembolic risk. Although ACL reconstruction is frequently discussed as a single clinical entity, surgical complexity may substantially modify the risk profile. Concomitant meniscal repair, multiligament reconstruction procedures involving the PCL, MCL, ALL, or posterolateral corner, prolonged operative time, extensive soft-tissue dissection, and prolonged postoperative non-weight-bearing protocols may increase thrombotic risk through greater tissue trauma and delayed mobilization. However, current evidence remains limited, and most international guidelines do not provide specific recommendations for these subgroups. Future studies should investigate whether thromboprophylaxis strategies should differ between isolated ACL reconstruction and more complex ligamentous procedures.

A significant consideration is the association between thromboprophylaxis and postoperative complications, particularly arthrofibrosis. Gu et al., analyzing data from 216,147 patients, demonstrated that the use of LMWH or DOACs was associated with an approximately twofold increased risk of MUA within two years postoperatively [8]. Furthermore, a systematic review by Hopper et al. identified anticoagulant use as an independent risk factor for MUA or lysis of adhesions (LOA), along with age, female sex, and concomitant meniscal repair [23].

Although pharmacological thromboprophylaxis has been associated with an increased risk of arthrofibrosis following ACL reconstruction, this association does not appear to be present when aspirin is used. Aspirin has been shown to provide similar efficacy to LMWH in preventing VTE in low-risk patients [5,24,25].

Zhu et al., in a systematic review, reported that LMWH was associated with a higher incidence of major bleeding events compared with no prophylaxis or control groups [26]. Therefore, the potential benefits of thromboprophylaxis must be carefully weighed against the risk of complications, particularly given the impact of arthrofibrosis on functional recovery.

Despite the general agreement against universal thromboprophylaxis and the emphasis on risk stratification, some studies have reported inconsistent associations between established risk factors and the occurrence of VTE following ACL reconstruction [20]. These findings highlight the limitations of current risk stratification models and suggest that VTE risk may not be fully explained by conventional clinical factors alone [27].

Emerging evidence indicates that genetic predisposition, including polymorphisms in specific loci, may play a role in individual thrombotic risk and could contribute to the heterogeneity observed across studies [27]. Incorporating such factors into future risk assessment models may improve the accuracy of patient stratification and guide more personalized thromboprophylaxis strategies.

Future studies should focus on ACLR through well-designed prospective studies and randomized controlled trials targeting high-risk subgroups rather than unselected patient populations. Large national registries and multicenter collaborations may play a crucial role in data collection on venous thromboembolism incidence, prophylaxis practices, and postoperative complications. Such approaches bring into line with the principles of precision medicine and may lead to more personalized and evidence-based thromboprophylaxis strategies following ACL reconstruction.

In summary, the existing evidence from international guidelines demonstrates a shift from universal prophylaxis toward a personalized, risk-based approach. While some healthcare systems continue to advocate routine prophylaxis, the majority of scientific organizations support risk-stratified protocols.

The orthopaedic surgeon plays a fundamental role in evaluating individual risk and selecting the most appropriate preventive strategy, balancing efficacy, safety, and patient-specific factors, and should be fully aware of the risk factors predisposing patients undergoing ACLR to DVT. Risk stratification tools, such as the Caprini score, can support clinical decision-making [17]. Early postoperative mobilization remains critical, as it may reduce the need for thromboprophylaxis while minimizing the risk of arthrofibrosis.

### 4.1. Proposed Clinical Algorithm Based on Current International Guidelines, Consensus Statements, and Registry-Based Recommendations

Although international guidelines differ in their recommendations regarding thromboprophylaxis after ACL reconstruction, several common principles emerge. Most contemporary guidelines support individualized risk assessment rather than universal pharmacological prophylaxis. Based on the available evidence and the recommendations of CHEST, NICE, BOA/BASK, ICM-VTE, ICS, and HAOST, a practical risk-stratified approach can be proposed.

In low-risk patients (e.g., young individuals without previous VTE, thrombophilia, obesity, hormonal therapy, prolonged immobilization, or significant comorbidities), early mobilization alone appears sufficient, and routine pharmacological thromboprophylaxis is generally not justified.

In moderate-risk patients, particularly those presenting one or more recognized risk factors but not fulfilling criteria for very high thromboembolic risk, mechanical prophylaxis and consideration of aspirin may represent a reasonable strategy, balancing VTE prevention against the risk of postoperative stiffness and bleeding.

In high-risk patients, including those with previous VTE, known thrombophilia, multiple cumulative risk factors, prolonged postoperative immobilization, or elevated Caprini scores, pharmacological thromboprophylaxis with LMWH or a DOAC should be strongly considered, alongside mechanical preventive measures and early mobilization.

**Figure 1:**
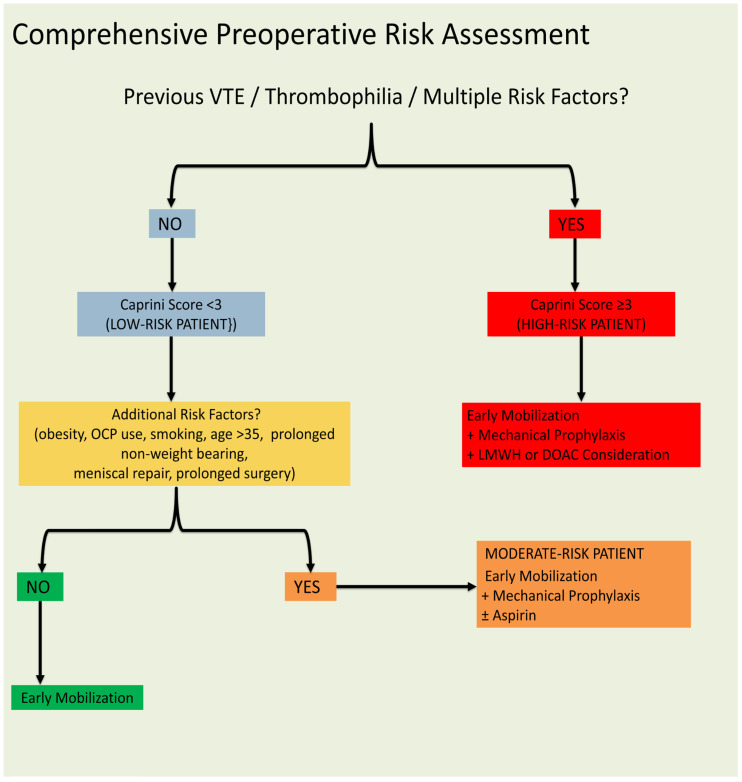
Proposed risk-stratified approach based on current evidence and international guidelines from CHEST, NICE, BOA/BASK, ICM-VTE, ICS, and HAOST. This algorithm represents a synthesis of current evidence and should not replace formal guideline recommendations. Risk stratification is based on current guideline recommendations and extrapolation from broader surgical populations, as ACL-specific validation of the Caprini score remains limited.

This proposed framework does not replace formal guideline recommendations but provides a pragmatic synthesis of the currently available evidence included in this manuscript and may assist clinicians when managing patients undergoing ACL reconstruction. A schematic summary of the proposed risk-stratified approach is presented in Figure 1.

### 4.2. Limitations

This review has several limitations. First, it was designed as a narrative review focused on international guidelines, consensus statements, and registry-based recommendations rather than as a systematic review conducted according to PRISMA methodology. Consequently, not all available studies evaluating thromboprophylaxis after ACL reconstruction were included. Second, guideline recommendations may not always fully reflect the most recent evidence, particularly in a rapidly evolving field. Nevertheless, the purpose of this review was to provide clinicians with a structured overview of current international practice patterns and areas of agreement and disagreement among major professional organizations.

## 5. Conclusions

Current evidence does not support routine pharmacological thromboprophylaxis for all patients undergoing ACL reconstruction. Despite variations among international guidelines, a common trend toward individualized risk assessment is evident.

Based on the available literature, a practical risk-stratified approach can be proposed. Low-risk patients may be managed with early mobilization alone, moderate-risk patients may benefit from aspirin and/or mechanical prophylaxis, whereas high-risk patients should be considered for pharmacological thromboprophylaxis with LMWH or DOACs in addition to mechanical preventive measures.

The Caprini Risk Assessment Model, although not specifically validated for ACL reconstruction, remains the most frequently recommended tool for guiding clinical decision-making. Particular attention should be given to patients with previous VTE, thrombophilia, obesity, prolonged immobilization, hormonal therapy, advanced age, or multiple cumulative risk factors.

It should also be noted that asymptomatic thrombotic events detected in screening studies suggest that the true thromboembolic risk following ACLR may be underestimated when only symptomatic events are considered and might be higher in routine clinical practice.

Future prospective studies and ACL-specific randomized trials are required to validate risk-stratification models and establish universally accepted thromboprophylaxis protocols for this unique patient population.

## Data Availability

No new data were created or analyzed in this study. Data sharing is not applicable to this article.

## Abbreviations

The following abbreviations are used in this manuscript:

ACL	Anterior Cruciate Ligament
ACLR	Anterior Cruciate Ligament Reconstruction
VTE	Venous Thromboembolism
DVT	Deep Vein Thrombosis
PE	Pulmonary Embolism
LMWH	Low-Molecular-Weight Heparin
DOAC	Direct Oral Anticoagulant
IPC	Intermittent Pneumatic Compression
ICS	International Consensus Statement
ICM-VTE	International Consensus Meeting on Venous Thromboembolism
HAOST	Hellenic Association of Orthopaedic Surgery and Traumatology
BOA	British Orthopaedic Association
BASK	British Association for Surgery of the Knee
BOSTAA	British Orthopaedic Sports Trauma and Arthroscopy Association
SFAR	Société Française d’Anesthésie et de Réanimation
AAOS	American Academy of Orthopaedic Surgeons
NICE	National Institute for Health and Care Excellence
CHEST	American College of Chest Physicians
